# Effects of Wuqinxi in the Patients with Chronic Low Back Pain: A Randomized Controlled Trial

**DOI:** 10.1155/2020/1428246

**Published:** 2020-08-18

**Authors:** Chongjie Yao, Zhenrui Li, Shuaipan Zhang, Zhiwei Wu, Qingguang Zhu, Lei Fang

**Affiliations:** ^1^Yueyang Hospital of Integrated Traditional Chinese and Western Medicine, Shanghai University of Traditional Chinese Medicine, No. 110 Ganhe Road, Hongkou District, Shanghai 200437, China; ^2^Research Institute of Tuina, Shanghai Academy of Traditional Chinese Medicine, No. 110 Ganhe Road, Hongkou District, Shanghai 200437, China; ^3^School of Rehabilitation Science, Shanghai University of Traditional Chinese Medicine, No. 1200 Cai Lun Road, Pudong New District, Shanghai 201203, China

## Abstract

Low back pain (LBP) is one of the major concerns of the current health care. The guidelines for chronic LBP recommend traditional Chinese exercise as an effective treatment. As one of the representatives of traditional Chinese exercise, Wuqinxi has been famous in China for its effects on improving health and treating chronic diseases for thousands of years. The objectives of the study were to assess the effects of Wuqinxi in the patients with chronic LBP on pain intensity, trunk muscle strength, and quality of life. The primary outcome measure was assessed by the Short-Form McGill Pain Questionnaire (SF-MPQ), including the Visual Analog Scale (VAS) and Present Pain Intensity (PPI) as the subtables. The effects of Wuqinxi on the quality of life were also assessed by the Short-Form Health Survey (SF-36) and the Pittsburgh Sleep Quality Index (PSQI) from physical component summary (PCS), mental component summary (MCS), and sleep quality. Besides, the electrical activities of the rectus abdominis (RA), obliquus externus abdominis (OEA), lumbar erector spinae (ES), and multifidus (MF) were assessed by integrated electromyogram (iEMG) after the end of the intervention. Both the groups showed statistically significant improvement in SF-MPQ, SF-36, PSQI, and iEMG at 12 weeks and 24 weeks when compared with baseline (*P* < 0.05). However, Wuqinxi demonstrated better effects in SF-MPQ and MCS after 24 weeks of intervention compared with the general exercise (*P* < 0.05). The patients in the Wuqinxi group (WQXG) also showed a significantly higher iEMG on OEA than the general exercise group (GEG) in 30°/s and 90°/s (*P* < 0.05). Our results showed that Wuqinxi had better effects on chronic LBP for a long time compared with general exercise, including pain intensity and quality of life. Thus, Wuqinxi should be recognized as a possible standalone therapy and self-management skill in chronic LBP, which is suitable for long-term practice.

## 1. Introduction

Low back pain (LBP) is one of the most common, costly, and disabling health conditions and now seems to be extending worldwide [1–3]. However, the symptoms are not attributed to particular etiologic or neurologic causes in 85% of the LBP patients, and most patients will not benefit from a single surgery [4]. So, patients with chronic LBP are eager to seek care and health services to relieve their pain [5]. A study showed that health care utilization due to chronic LBP was increasing every year, but the number of patients with disability increased by 54% from 1990 to 2015 [6].

The causes of chronic LBP are quite complicated, which may be attributed to long-term excessive physical exertion or trauma, resulting in strain or degradation of the vertebrae, intervertebral discs, or spinal muscles [7]. Therefore, patients with chronic LBP should also be treated for a period of time to achieve long-term results. However, the financial burden will increase with the frequent use of spinal injections, analgesic drugs, or visits to therapists [8].

Current guidelines for chronic LBP recommend exercise as an effective treatment option [9, 10]. Past studies have shown that exercise therapy appears to be effective in relieving pain and improving physical function, which encompasses various interventions, ranging from aerobic exercises to muscle strengthening and stretching [11–13]. On the one hand, exercise therapy can strengthen and train the core muscles, so as to enhance the stability of the body [14]; on the other hand, breathing control during exercise is also important for the rehabilitation of chronic LBP because it plays a role in exercising the core muscles, such as the diaphragm and abdominal internal and external oblique muscles [15]. However, many forms of physical activity are either very intense or monotonous, making it difficult to practice and keep for a long time. Traditional Chinese exercise has long been used to raise the physical and moral integrity level and prevent chronic disease progression [16]. Tai-chi exercise is one of the traditional Chinese exercises, which is recommended as a therapeutic exercise in the guidelines of the American College of Physicians because of its good effect on chronic LBP [10].

Wuqinxi (five-animal exercise) is another famous traditional Chinese exercise, which was created by a well-known Chinese physician Huatuo in Donghan Dynasty. It mimics the movements of five animals in terms of tiger, deer, bear, monkey, and crane, which emphasizes the integration of body movement, breathing, and mental together harmoniously during practice. Therefore, compared with Tai-chi exercise, Wuqinxi pays more attention to the relationship between human and nature. With the enhancement of people's awareness of self-care, more and more studies have shown the benefits of Wuqinxi in improving health and treating chronic diseases [17–19]. A prospective study demonstrated that Wuqinxi can improve the function of the lumbosacral multifidus and reduce LBP, but no further efficacy evaluation was carried out [20]. Therefore, the objectives of the study were to assess the effects of Wuqinxi in patients with chronic LBP at the end of a 24-week treatment program on pain intensity, trunk muscle strength, and quality of life.

## 2. Methods

### 2.1. Study Design

This study was a parallel-group, assessor- and analyst-blinded randomized controlled trial (RCT) conducted in the Department of Physiotherapy, Shanghai Hongkou District Quyang Community Health Service Center, and the Yueyang Hospital of Integrated Traditional Chinese and Western Medicine for 24 weeks. Eligible participants were randomly assigned to the Wuqinxi group (WQXG) and the general exercise group (GEG) in a 1 : 1 ratio. The study protocol was in accordance with the Declaration of Helsinki and was approved by the Chinese Ethics Committee (No. ChiECRCT-20160048). Also, we registered the study on the Chinese Clinical Trial Registry (No. ChiCTR-INR-16009038). The sample size calculation of the trial was based on our previous study, with the power of 0.9 (1-*β*) and a significant *P* value less than 0.05. Taking a possible 15% drop rate into consideration, a total of 72 participants were enrolled.

### 2.2. Participant Recruitment

The patients with chronic LBP consistent with the disease definition in the ICD-11 were recruited. The 72 participants were recruited from the Shanghai Hongkou District Quyang Community Health Service Center and the Yueyang Hospital of Integrated Traditional Chinese and Western Medicine. We also sought a few patients through posters, Internet advertisements, and the official microblog and WeChat platforms. According to guidelines in [21], chronic LBP is defined as pain and discomfort persisting for at least 12 weeks, localized below the costal margin and above the inferior gluteal folds, with or without referred leg pain.

#### 2.2.1. Inclusion Criteria

The inclusion criteria were as follows:

1. History of LBP as the main symptom persisting for at least 3 months
2. Pain lasting for more than 20 min per time and at least once per month
3. Aged between 18 and 70 years, male or female
4. Promise not to receive other related therapy (e.g., analgesic drugs) during the period of treatment
5. Volunteer to take part in the study and sign the informed consent form

#### 2.2.2. Exclusion Criteria

The exclusion criteria were as follows:

1. Caused by specific diseases (fractures, carcinoma, anomalies, disc prolapse, spinal stenosis, tumor, spinal infection, ankylosing spondylosis, spondylolisthesis, kyphosis or structural scoliosis, and nerve root affection with neurological signs)
2. Severe primary diseases such as cardiovascular, lung, kidney, and hematopoietic disease and mental disorder
3. Surgery to the low back within the past 6 months
4. Received formal physical therapy or other therapies in the last 1 month
5. Pregnant or lactating women

#### 2.2.3. Dropout and Suspension Criteria

According to the Patient Management Protection Rules, patients have the right to withdraw for any reason during the study period. The following conditions were considered as withdraw criteria:

1. Patients cannot finish the protocol treatment on schedule
2. Participation in other treatments during the trial
3. Intolerable adverse events
4. Lost to follow-up

In addition, if severe poor efficacy or adverse reactions had occurred during the trial, the trial was forcibly suspended immediately.

### 2.3. Randomization and Allocation

Eligible participants were randomly assigned to the WQXG and GEG, with 36 patients in each group. The randomization sequence was computer generated and concealed in sealed, opaque envelopes by a member of the research team not involved in recruitment. The therapist was responsible for sequentially opening randomly assignment envelopes and allocating the participants accordingly.

### 2.4. Blinding

Owing to the limitation of the study, participants and therapists were not possible to be blinded to treatment. For the sake of reducing the risk of bias, we told the patients that the purpose of the study was to compare the two exercise therapies, and the patients were unlikely to know which group they were in. In addition, the evaluators, data managers, and statisticians were blinded to the group allocation in the outcome evaluation procedure and data analysis.

### 2.5. Interventions

Patients from both groups participated in a supervised exercise therapy program four times a week with 1 hour of each session for 24 weeks. Heart rate changes and rating of perceived exertion (RPE) were recorded to keep the same volume of exercise [22]. All the participants were called to the Quyang Community Health Service Center to take part in exercise training and a short forum of back pain before starting the intervention. The therapist must have 10 years of experience of the traditional Chinese exercises and be skillful in health care education. The standard movements of the therapist were recorded as videos and distributed to patients to guide their future treatment. Dedicated logbooks were sent to patients to record the weekly exercise and supervised by our researchers on WeChat. Besides, guidance was given outdoors every month, which also helped us evaluate the recovery of everyone.

#### 2.5.1. Intervention Methods in the WQXG

The patients participated in a supervised Wuqinxi program for 24 weeks. The sessions were conducted four times a week with 1 hour of each session. Each session consisted of 10 minutes of warm-up, 40 minutes of Wuqinxi, and 10 minutes of cool down. Wuqinxi imitates the specific movements of five animals in terms of tiger, deer, bear, monkey, and crane, which combines breathing control, body movement, and meditation. The core movements of Wuqinxi are illustrated in Table 1. At the beginning of each month, patients in this group were called to exercise and receive guidance from the therapist.

#### 2.5.2. Intervention Methods in the GEG

The patients participated in a supervised exercise program with the same volume of training as Wuqinxi for 24 weeks. The exercise included the movements addressing muscle activity of the abdominals, erector spinae, gluteal, quadriceps, and hamstrings muscle groups. The exercise program started with nonweight bearing positions and progressed by increasing load. Weights and resistance were individualized according to the physical capacity of the patient and progressively increased according to the guidelines of the American College of Sports Medicine [23]. This exercise was chosen as the control intervention because it is credible for the treatment of LBP [24]. At the end of each month, patients in this group were called to exercise and receive guidance from the therapist.

### 2.6. Outcome Measurements

The outcomes were measured at 12 weeks and 24 weeks, which can reflect the changes in pain intensity, quality of life, and trunk muscle strength of the patients with chronic LBP.

#### 2.6.1. Primary Outcome Measurement

The primary outcome measure was assessed by the Short-Form McGill Pain Questionnaire (SF-MPQ), which has scores ranging from 0 to 45 [25]. The higher SF-MPQ values indicated greater pain levels. The Visual Analog Scale (VAS) as the subtable of SF-MPQ was used to grade the pain severity of patients by a 10-point scale, where 0 means no pain and 10 means severe or unbearable pain [26]. Another subtable of SF-MPQ was Present Pain Intensity (PPI), which has a 6-point scale ranging from 0 (no pain) to 5 (unbearable pain). PPI was used for the measurement of the intensity of pain at the time of the evaluation [23].

#### 2.6.2. Secondary Outcome Measurement

Secondary outcome measures included the Short-Form Health Survey (SF-36) and the Pittsburgh Sleep Quality Index (PSQI). The SF-36 had been applied and validated several times for intervention studies with back pain [27, 28]. The questions were divided into eight domains, which combined physical and psychological questions. Domain scores may be aggregated and normalized using a standard algorithm into two summary component scores, physical component summary (PCS) and mental component summary (MCS), where a value of 50 represents the population norm and higher scores indicate better health status. PSQI was developed by Buysse et al. [29] in 1989 and has been used to assess sleep quality and sleep mode of individuals in the latest months. The qualities assessed included subjective sleep quality, sleep latency, sleep duration, sleep efficiency, sleep disorders, hypnotic agents, and daytime dysfunction (ranging from 0 to 21, with higher scores indicating worse sleep quality). The Chinese version of PSQI and the SF-36 scale was used in this study, and its reliability and validity have been verified in the Chinese population [30].

#### 2.6.3. The Measurement of Trunk Muscle Strength

In addition, trunk muscle strength was assessed by integrated electromyogram (iEMG) after the end of the intervention. A surface electromyography system (Telemyo 2400T-G2 Telemetry EMG system; Noraxon, USA) with disposable bipolar electromyography electrodes was used to measure the electrical activities of the rectus abdominis (RA), obliquus externus abdominis (OEA), lumbar erector spinae (ES), and multifidus (MF) with 1000 Hz sampling frequency [31]. The angular velocity of isokinetic concentric (flexion) and eccentric (extension) movement was set as 30°/s and 90°/s, and each complete movement was regarded as a cycle to repeat 10 times.

### 2.7. Statistical Analyses

SPSS 21.0 software was used for the analysis. The normality and homogeneity of variance tests were performed on the data. The data were presented as the mean ± standard deviation (SD) if they were normally distributed; otherwise, they were presented as the median (*P*25 and *P*75). The changes in mean were calculated from the difference between the current value and the baseline data. Parametric statistics (independent samples *t*-test and paired samples *t*-test) or nonparametric statistics (Wilcoxon rank-sum test) were used for the within- and between-group analyses in accordance with the results of the homogeneity and normality analyses. When initial homogeneity and normality of data distribution are found, repeated measures analysis of variance (ANOVA) and ANOVA with adjustment of the Bonferroni correction were used to analyze within and between groups. The Friedman test and the Kruskal–Wallis test will be used when initial homogeneity but not the normality of data distribution is found. If the initial homogeneity is not found, a linear mixed model will be adjusted for the baseline value. The statistical significance was defined as *P* < 0.05, and the 95% confidence interval (CI) was reported.

## 3. Results

### 3.1. Recruitment and Baseline Data

**Figure 1:**
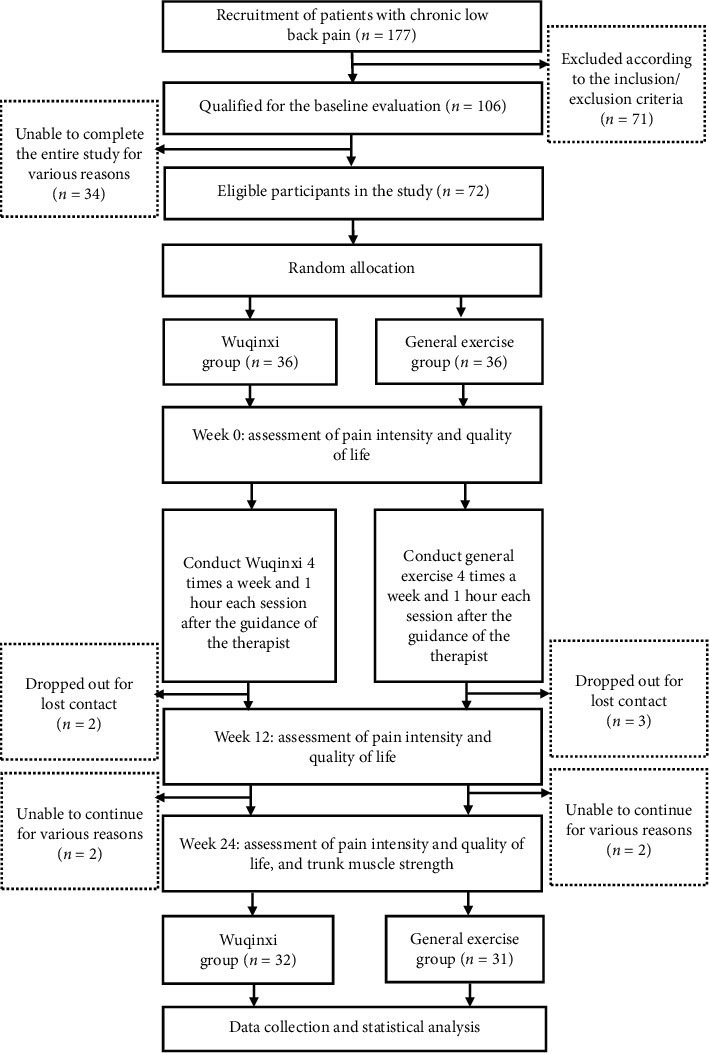
Flow diagram of the study.

Flow diagram of the study is presented in Figure 1. A total of 177 patients were recruited, but 71 patients did not meet the inclusion criteria. 106 patients were qualified for the baseline evaluation, among which 34 were unable to complete the entire study for various reasons. The remaining 72 patients met the eligibility criteria and signed the informed consent form. Of the 72 eligible participants, 67 completed the assessment at 12 weeks, and 2 in the WQXG and 3 in GEG dropped out as contact was lost after several weeks of exercise. At 24 weeks, 63 participants finally completed the assessment because 2 participants in each group withdrew from the study after 12 weeks of intervention. Participants in the WQXG and GEG completed 89% and 86% of the total planned exercise session, respectively.

The baseline demographic and clinical characteristics of all participants in both groups are shown in Table 2. The recruited population had a mean age of 53.4 years with a female predominance. The mean body mass index (BMI) was in the normal range, and the participants had LBP for 12.8 years. No significant differences were found between groups (*P* > 0.05) on demographics, medical history, quality of life, and pain intensity at baseline.

### 3.2. Effects of Wuqinxi on Pain Intensity

**Figure 2:**
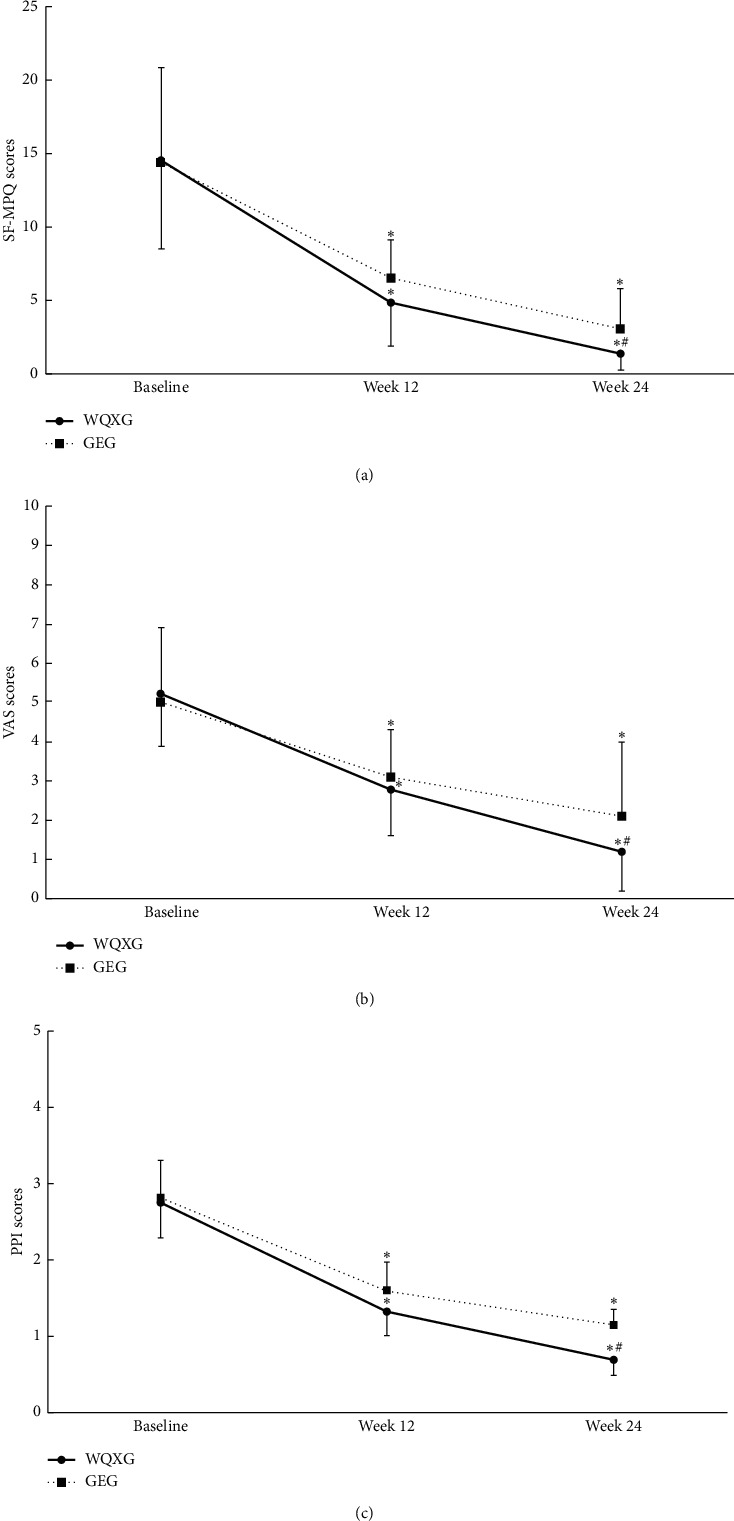
The changes in pain intensity from baseline to 12 and 24 weeks. The pain intensity was assessed by SF-MPQ, and VAS and PPI as subtables were used for further evaluation. (a) The changes in the SF-MPQ scores from baseline to 12 and 24 weeks. (b) The changes in the VAS scores from baseline to 12 and 24 weeks. (c) The changes in the PPI scores from baseline to 12 and 24 weeks. Compared with baseline, ^*∗*^*P* < 0.05; compared with the GEG at the same time point, ^#^*P* < 0.05. WQXG, Wuqinxi group; GEG: general exercise group; SF-MPQ: Short-Form McGill Pain Questionnaire; VAS: Visual Analog Scale; PPI: Present Pain Intensity.

The changes from baseline to 12 and 24 weeks in the two groups for pain intensity are shown in Table 3 and Figure 2 in general. At 12 weeks, WQXG and GEG, respectively, showed a significant decrease in the SF-MPQ total score, VAS, and PPI when compared with baseline (*P* < 0.05), but there was no significant difference between the groups (*P* > 0.05). At 24 weeks, the WQXG had a greater decrease in the SF-MPQ total score, VAS, and PPI than the GEG, and the difference was statistically significant (*P* < 0.05). The differences in the means of SF-MPQ, VAS, and PPI between the groups were −1.7 points (95% CI −3.2 to −0.2), −0.9 points (95% CI −1.8 to −0.1), and −0.6 points (95% CI −1.0 to −0.1) at 24 weeks, which indicated that Wuqinxi was more beneficial in relieving pain than the general exercise for patients with chronic LBP.

### 3.3. Effects of Wuqinxi on Quality of Life

The quality of life was assessed by the SF-36 and PSQI from physical function, mental health, and sleep quality. The changes in the SF-36 and PSQI scores from baseline to 12 and 24 weeks between groups are shown in Table 4 in general. WQXG and GEG, respectively, showed a significant improvement in physical and mental component scores at 12 weeks compared with baseline (*P* < 0.05), but there was no statistically significant difference between groups (*P* > 0.05). At 24 weeks, compared with the GEG, the WQXG had a greater improvement in the scores of the SF-36 physical component and the mental component, but only the difference in MSC was statistically significant (*P* < 0.05). The difference in the mean MSC between groups was 6.7 points (95% CI, 0.8 to 12.5), which indicated a better mental health in the WQXG. In addition, PSQI scores of the two groups at week 12 and week 24 were significantly lower than that at baseline (*P* < 0.05), but the difference between the WQXG and GEG at the same time point was not statistically significant.

### 3.4. Effects of Wuqinxi on Trunk Muscle Strength

The electrical activities of RA, OEA, ES, and MF were measured to assess trunk muscle strength by iEMG after the end of the intervention. The effects of the 2 different exercise interventions on the lumbar and absolute muscles are shown in Table 5. In our results, both five-animal exercise and general exercise were beneficial to ES, but there were no statistically significant differences (*P* > 0.05). The two exercise interventions improved RA, MF in 30°/s, and MF in 90°/s, and the differences were statistically significant (*P* < 0.05). However, there was no significant difference between the two groups in the improvement of RA and MF. Besides, it was found that general exercise may have no effect on OEA because the electrical activity change of OEA in 30°/s was −2.2 (−3.6 to −0.9) compared with baseline. In contrast, the effect of Wuqinxi on OEA was better than that of general exercise whether at 30°/s or 90°/s, and the difference was statistically significant (*P* < 0.05).

## 4. Discussion

### 4.1. Analysis of the Research Results

The traditional Chinese exercise can induce a relaxed state of mind, which is different from the general exercise. Some techniques of traditional Chinese exercise guide and transfer attention from pain via meditating and imagining, which can help alleviate back pain, improve psychosocial well-being, and increase confidence. As a representative, the Tai-chi exercise has attracted attention worldwide in the field of sports medicine for its good effect on chronic diseases [32–34]. However, Wuqinxi has become an alternative choice in China in recent years due to its unique movements and effects [35]. Wuqinxi is a mind-body exercise integrated with training for body strength, flexibility, breathing, and meditating, which emphasizes the unification of mental regulation, breathing exercises, and movement control [35]. Patients need to imagine that they are imitating the might of tiger, vigor of deer, steadiness of bear, flexibility of monkey, and stretching movement of crane. A meta-analysis demonstrated that Wuqinxi significantly improved pain symptom and lumbar spine bone mineral density compared with antiosteoporosis medications, indicating the effect of Wuqinxi on pain [19].

SF-MPQ score is a validated measure for the assessment of acute pain, chronic pain, and postoperative pain, including VAS and PPI as subtables [36]. The findings of our study suggested that both Wuqinxi and general exercise resulted in good effects on chronic pain, which were consistent with the results of published clinical studies [37, 38]. Compared with the GEG, the patients in the WQXG showed a better effect in reducing pain at 24 weeks, and the difference was statistically significant (*P* < 0.05). It might be related to the greater advantage of Wuqinxi in improving related muscle groups. iEMG was used to reflect the total discharge of the motor units participating in the movement at a certain time and was expressed as the integral of the area surrounded by the myoelectricity changing curve and the horizontal axis of time [39]. Nahhas Rodacki et al. [40] suggested that abdominal exercise was associated with the improvement in LBP since the pressure on the intervertebral disks was decreased as a consequence of the increased intra-abdominal pressure during abdominal contraction. However, the strength of OEA was decreased after general exercise in our results, which may be related to insignificant efficacy and development of the disease. Wuqinxi emphasizes on the coordination of breathing and movements, especially training on the breathing pattern, which may stretch OEA and other abdominal at deep inspiration training. The above may be the reason for the better effect of Wuqinxi than the general exercise on pain.

We also observed the effects of Wuqinxi on the quality of life, which were assessed by the SF-36 and PSQI from physical function, mental health, and sleep quality. Our study showed that the PCS and MCS in SF-36 of the participants were significantly improved in the WQXG and GEG at 24 weeks compared with the baseline. However, compared with the general exercise, Wuqinxi has a better effect on mental health, and the difference is statistically significant (*P* < 0.05). A large number of studies confirmed that qigong was more beneficial to psychology than general exercise, which was also consistent with our research results [41–43]. Besides, the patients with chronic LBP in the WQXG or GEG had a significant improvement on the sleep quality of at 24 weeks compared with baseline, according to the changes in PSQI scores. However, there was no statistical difference between the two groups, which may need to be further confirmed through a large-sample study.

### 4.2. Limitations of the Study

First of all, as a prospective experiment, the sample size of the study needs to be improved. In the future, multicenter and large-sample studies should be carried out to determine the therapeutic effect of Wuqinxi on chronic LBP. Secondly, the study lasted a long time and depended on the self-management of patients. Although we communicated with the patients every week and gave them instruction every month, we still cannot guarantee their completion of the experiment. Also, although it can be learned from the network system of the hospital whether the patients received other related treatments in the past 24 weeks, we still cannot neglect the possibility of purchasing drugs from drugstores. But, as a reward, we promised to give free physical therapy for the patients who seriously complete the whole experiment if they have other problems in the future. Therefore, the completion of the study was largely dependent on the active cooperation of patients.

## 5. Conclusion

The above results showed that Wuqinxi had better effects on chronic LBP for a long time compared with the general exercise, including pain intensity and quality of life. Thus, Wuqinxi should be recognized as a possible standalone therapy and self-management skill in chronic LBP, which is suitable for long-term practice. Further multicenter large-sample studies should be designed to compare Wuqinxi with other exercises to discover the advantages of traditional Chinese exercise in the treatment of chronic LBP. Besides, strategies are needed to increase motivation for the regular practice of Wuqinxi and explore the possibility of self-management skills in chronic diseases.

## Figures and Tables

**Table 1:** The core movements of Wuqinxi.

Tiger movement	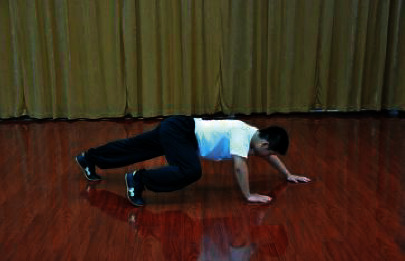	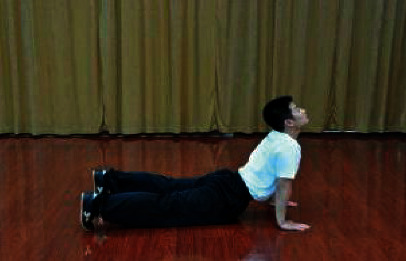
Deer movement	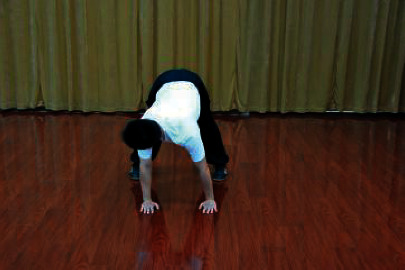	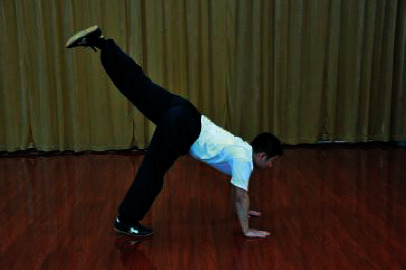

Bear movement	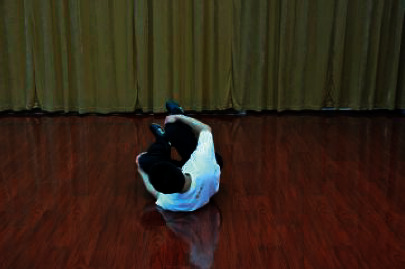	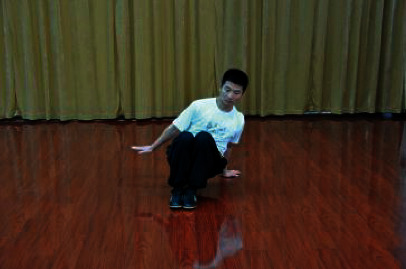

Monkey movement	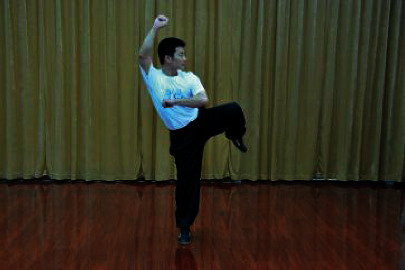	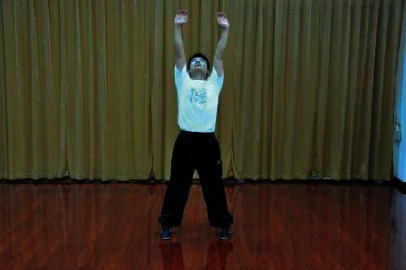

Crane movement	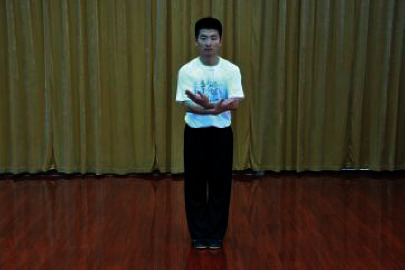	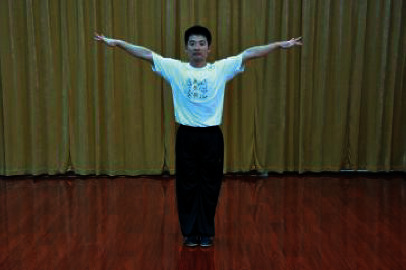

**Table 2:** The baseline demographic and clinical characteristics of participants (mean ± SD).

Demographic characteristic	WQXG (*n* = 36)	GEG (*n* = 36)
Age (year)	53 (16)	54 (14)
Gender	30 (83)	28 (78)
Male	6 (17%)	8 (22%)
Female	30 (83%)	28 (78%)
Height (cm)	161 (6.3)	163 (5.8)
Weight (kg)	61 (7.9)	63 (10.6)
Received education years (year)	14 (3.2)	14 (2.8)
Duration of LBP (year)	13.2 (9.2)	12.4 (10.8)
BMI	23.4 (3.1)	23.6 (3.5)
Outcome measures		
SF-MPQ	14.5(6)	14.4 (6.5)
VAS	5.2 (1.3)	5 (1.9)
PPI	2.7 (0.9)	2.8 (1.1)
PSQI	8.8 (4.4)	9.7 (5.2)
PCS	34.6 (8.9)	34.4 (8.8)
MCS	34.1 (11.9)	37.5 (12.8)

There were no significant differences between groups on demographics, medical history, quality of life, and pain intensity at baseline (*P* > 0.05). SD, standard deviation; WQXG, Wuqinxi group; GEG: general exercise group; LBP, low back pain; BMI: body mass index; SF-MPQ: Short-Form McGill Pain Questionnaire; VAS: Visual Analog Scale; PPI: Present Pain Intensity; PSQI: Pittsburgh Sleep Quality Index; PCS: mental component summary; MCS: physical component summary.

**Table 3:** The changes in pain intensity from baseline to 12 and 24 weeks.

Outcomes	Difference in mean change (95% CI)		Between-group difference (95% CI)	
	WQXG	GEG	GEG vs. WQXG	*P*
SF-MPQ (0–45)				
Week 12	−9.8 (−11.9 to −7.6)^*∗*^	−7.9 (−10.4 to −5.3)^*∗*^	−1.7 (−3.5 to 0.01)	0.051
Week 24	−13.3 (−15.4 to −11.2)^*∗*^	−11.4 (−13.9 to −8.9)^*∗*^	−1.7 (−3.2 to −0.2)^#^	0.032

VAS (0–10)				
Week 12	−2.5 (−3.2 to −3.8)^*∗*^	−1.9 (−2.8 to −1.0^*∗*^	−0.3 (−1.0 to 0.4)	0.357
Week 24	−4.0 (−4.7 to −3.4)^*∗*^	−2.8 (−3.8 to −1.9)^*∗*^	−0.9 (−1.8 to −0.1)^#^	0.035

PPI (0–5)				
Week 12	−1.4 (−1.8 to −1.0)^*∗*^	−1.2 (−1.7 to −0.7)^∗^	−0.3 (−0.7 to 0.1)	0.166
Week 24	−2.1 (−2.5 to −1.7)^*∗*^	−1.6 (−2.1 to −1.1)^*∗*^	−0.6 (−1.0 to −0.1)^#^	0.014

The pain intensity was assessed by SF-MPQ, and VAS and PPI as subtables were used for further evaluation. The changes in mean were calculated from the difference between the current value and the baseline data. Compared with baseline, ^*∗*^*P* < 0.05; compared with the GEG at the same time point, ^#^*P* < 0.05. CI, confidence interval; WQXG, Wuqinxi group; GEG: general exercise group; SF-MPQ: Short-Form McGill Pain Questionnaire; VAS: Visual Analog Scale; PPI: Present Pain Intensity.

**Table 4:** The changes in the quality of life from baseline to 12 and 24 weeks.

Outcomes	Difference in mean change (95% CI)		Between-group difference (95% CI)	
	WQXG	GEG	GEG vs. WQXG	*P*
SF-36 PCS				
Week 12	13.6 (7.7 to 19.4)^*∗*^	12.6 (3.3 to 21.9)^*∗*^	0.7 (-5.2 to 3.8)	0.051
Week 24	34.0 (28.2 to 39.9)^*∗*^	24.0 (14.7 to 33.3)^*∗*^	3.1 (-1.8 to 8.0)	0.032

SF-36 MCS				
Week 12	12.1 (5.7 to 18.6)^*∗*^	8.5 (−0.9 to 17.9)^*∗*^	1.5 (−5.4 to 8.4)	0.665
Week 24	29.8 (23.3 to 36.3)^*∗*^	16.7 (7.3 to 26.1)^*∗*^	6.7 (0.8 to 12.5)^#^	0.028

PSQI				
Week 12	−2.8 (−4.7 to −0.9)^*∗*^	−2.7 (−4.6 to −0.9)^*∗*^	−0.6 (−2.2 to 1.0)	0.451
Week 24	−3.1 (−5.2 to −1.0)^*∗*^	−3.9 (−5.9 to −1.9)^*∗*^	0.3 (−1.3 to −2.0)	0.681

The quality of life was assessed by the SF-36 and PSQI from physical function, mental health, and sleep quality. The changes in mean were calculated from the difference between the current value and the baseline data. Compared with baseline, ^*∗*^*P* < 0.05; compared with the GEG at the same time point, ^#^*P* < 0.05. CI, confidence interval; WQXG, Wuqinxi group; GEG: general exercise group; SF-36, Short-Form Health Survey; PCS, physical component summary; MCS, mental component summary; PSQI, Pittsburgh Sleep Quality Index.

**Table 5:** The changes in electrical activities of trunk muscle strength from baseline to 24 weeks.

Muscle	Difference in mean change (95% CI)		Between-group difference (95% CI)	
	WQXG	GEG	GEG vs. WQXG	*P*
RA				
30°/s	10.3(1.7 to 18)^*∗*^	11.7(1.9 to 21.5)^*∗*^	−1.4(−13 to 10.2)	0.78
90°/s	2.1(−14.1 to 18.2)	7.4(−2.3 to 17.1)	−5.3(−14.1 to 9.3)	0.64

OEA				
30°/s	8.2(4.3 to 12.1)^*∗*^	−2.2(−3.6 to −0.9)^*∗*^	10.4(4.3 to 24.4)^#^	0.048
90°/s	3.0(−9.8 to 15.9)	−7.5(−22.3 to 7.3)	10.5(1.9 to 19.1)^#^	0.045

ES				
30°/s	−1.6(−16.6 to 13.4)	3.2(−6.7 to 33.2)	−4.8(−24.7 to 10.1)	0.24
90°/s	3.3(−7.7 to 14.3)	7.8(−8.7 to 22.8)	−4.5(−19.6 to 10.7)	0.69

MF				
30°/s	8.4(2.9 to 13.9)^*∗*^	15.4(1.9 to 38.9)^*∗*^	−7(−17.8 to 4.3)	0.34
90°/s	21.3(4.4 to 38.2)^*∗*^	9.1(3.3 to 14.9)^*∗*^	12.2(−10.7 to 35.1)	0.42

The electrical activities of RA, OEA, ES, and MF were measured in the angular velocity of 30°/s and 90°/s, respectively, to assess trunk muscle strength after the end of the intervention. The changes in mean were calculated from the difference between the current value and the baseline data. Compared with baseline, ^*∗*^*P* < 0.05; compared with the GEG at the same time point, ^#^*P* < 0.05. CI, confidence interval; WQXG, Wuqinxi group; GEG: general exercise group; RA, rectus abdominis; OEA, obliquus externus abdominis; ES, erector spinae; MF, multifidus.

## Data Availability

The data used to support the findings of this study are available from the corresponding author upon request. He can be reached at fanglei586@126.com.

## Abbreviations

LBP: Low back pain
WQXG: Wuqinxi group
GEG: General exercise group
SF-MPQ: Short-Form McGill Pain Questionnaire
VAS: Visual Analog Scale
PPI: Present Pain Intensity
SF-36: Short-Form Health Survey
PSQI: Pittsburgh Sleep Quality Index
iEMG: Integrated electromyogram
PCS: Physical component summary
MCS: Mental component summary
RA: Rectus abdominis
OEA: Obliquus externus abdominis
ES: Erector spinae
MF: Multifidus
ANOVA: Analysis of variance
CI: Confidence interval.
